# Case Report: Reduced CSF Orexin Levels in a Patient With Sepsis

**DOI:** 10.3389/fnins.2021.739323

**Published:** 2021-10-06

**Authors:** Yasuhiro Ogawa, Seioh Ezaki, Nobutake Shimojo, Satoru Kawano

**Affiliations:** Department of Internal Medicine, University of Tsukuba Hospital, Tsukuba, Japan

**Keywords:** orexin, sepsis, cerebrospinal fluid, blood, blood-brain barrier

## Abstract

Sepsis is a potentially lethal condition characterized by systemic inflammation and multiple organ failure, and sepsis-associated encephalopathy (SAE) is an independent risk factor for mortality in patients with sepsis. We previously reported that orexin improved survival in an animal model of sepsis by acting in the brain. Peripherally administered orexin entered the brain under the conditions of systemic inflammation because of BBB dysfunction and produced survival-related effects. As a therapeutic concept, we hypothesized that orexin treatment enhances recovery from sepsis by restoring reduced orexin levels in cerebrospinal fluid (CSF). Here, we report that CSF orexin levels were reduced in a 63-year-old woman with sepsis. The patient presented with coma, fever, headache, vomiting, and seizures upon arrival at the emergency room. She had a history of subarachnoid hemorrhage which led to the development of hydrocephalus, and as a consequence, a ventriculoperitoneal shunt (VP shunt) tube had been installed to ameliorate the complication. Physical examinations showed dehydration and abnormality of circulation, arterial blood gas analysis showed insufficient oxygenation, blood tests showed an inflammatory response, liver injury, kidney injury, hyperkalemia, and hyperglycemia, and radio graphical examinations showed mild hydrocephalus and several old microinfarctions. She was diagnosed with sepsis because her Sequential Organ Failure Assessment (SOFA) score was 13 and *Enterococcus faecalis* was isolated form her blood and CSF. Status epilepticus, hyperglycemia, and sepsis-associated encephalopathy were considered possible causes of coma. Her CSF could be safely sampled because she had a VP shunt, although it is ethically difficult to sample CSF routinely from patients with sepsis. Reduced CSF orexin levels gradually recovered as she recovered from sepsis. Unexpectedly, orexin was detected in the blood, which is unusual in healthy humans. Blood orexin was not detected after recovery from sepsis. This result may imply that orexin leaks into the blood because of BBB dysfunction. To the best of our knowledge, this is the first report investigating orexin levels in the CSF and blood of a patient with sepsis, and the data obtained from this case may provide a new understanding of the pathophysiology of SAE.

## Introduction

Sepsis was first defined in 1993 as systemic inflammatory response syndrome (SIRS) due to infection, which is the initial condition in the pathophysiology of sepsis (Cohen, 1993; Young et al., 1993); sepsis was recently redefined as life-threatening organ dysfunction caused by a dysregulated host response to infection and is clinically correlated with mortality (Singer et al., 2016). As noted in these definitions, the pathophysiology of sepsis is characterized by systemic inflammation and subsequent multiple organ failure (Cohen, 2002), but the cellular and molecular mechanisms are very complex, and effective therapies based on the pathophysiology of sepsis are not yet available (Angus and van der Poll, 2013). Systemic inflammation damages not only the peripheral organs (e.g., lung, kidney, and heart) but also the central nervous system (CNS), which is called sepsis-associated encephalopathy (SAE). According to previous reports, blood-brain barrier (BBB) dysfunction plays a primary role in the pathophysiology of SAE. SAE is recognized as an independent risk factor for mortality in patients with sepsis and for prolonged cognitive impairment in patients who survive sepsis (Widmann and Heneka, 2014). Therefore, the CNS can be a novel therapeutic target for sepsis. However, the pathophysiology of SAE is not yet well understood.

The hypothalamic neuropeptide orexin regulates wakefulness/sleep by working in the brain; orexin deficiency causes narcolepsy, a sleep disorder (Sakurai, 2007). In healthy humans, orexin can be detected (range 250–500 pg/ml) in cerebrospinal fluid (CSF), but it is generally not detected (<40 pg/ml) in blood by a standard orexin radioimmunoassay, which is used for the diagnosis of narcolepsy, because it cannot cross the BBB. By using a novel specific and sensitive orexin radioimmunoassay, blood orexin can be detected at very low levels (range 0.5–16 pg/ml), but it does not correlate with the wake/sleep cycle (Makela et al., 2018). Surprisingly, we previously reported that orexin improved survival in mice with endotoxin shock, a well-studied animal model of sepsis, by acting in the brain (Ogawa et al., 2016), indicating that orexin may be involved in the pathophysiology of SAE and be a new agent in targeting the CNS for the treatment of sepsis. Interestingly, even peripherally administered orexin entered the brain under systemic inflammation because of BBB dysfunction and produced survival-related effects in mice with endotoxin shock (Ogawa et al., 2016). As the mechanism of action, we showed that orexin inhibited the excess inflammatory response and ameliorated hypothermia and bradycardia in mice with endotoxin shock by regulating the neuroendocrine and autonomic nervous systems under CNS control (Ogawa et al., 2016). Although the therapeutic concept remains unclear, we hypothesize that orexin treatment enhances recovery from sepsis by restoring reduced orexin levels in the CSF. However, CSF orexin levels in patients with sepsis have never been reported. Here, we report orexin levels in the CSF and blood of a patient with sepsis on the day of admission to the intensive care unit (ICU), 2 days after admission, on the day of transfer from the ICU to the general ward (GW), and 1 week after antibiotic treatment completion.

A 63-year-old woman presented with coma, fever, headache, vomiting, and seizures upon arrival at the emergency room. Initially, her quick Sequential Organ Failure Assessment (qSOFA) score was three (systolic blood pressure: 94 mmHg, respiratory rate: 24/min, Glasgow Coma Scale (GCS) score: six), according to the Sepsis-3 criteria (Table 1). Physical examinations showed coldness of limbs and dryness of skin and mouth but not trauma, abnormality of skin, nuchal rigidity, pupil abnormality, or paralysis of limbs. However, detailed neurological examinations could not be performed because of coma. The patient had a history of subarachnoid hemorrhage. A ventriculoperitoneal shunt (VP shunt) tube was installed over 20 years prior to the present episode to alleviate hydrocephalus which occurred as a complication of subarachnoid hemorrhage but had been non-functional for at least 10 years because of tube breakage. The patient was on an antihypertensive medication. After sampling her blood and CSF, we started an intravenous infusion of crystalloid solution (2400 ml/day) and norepinephrine (0.15 μg/kg/min), oxygen administration (6 L/min via mask), antibiotic treatment (ceftriaxone), and antiepileptic treatment (levetiracetam). CSF samples were collected from the VP shunt tube. Arterial blood gas analysis showed insufficient oxygenation (PaO_2_:78.4 mmHg under conditions of oxygen administration at 6 L/min via mask, PaO_2_-FiO_2_ ratio: 156.8, lactate: 1.1 mmol/L). Blood tests showed an inflammatory response (white blood cell count: 24,800 cells/μl, C-reactive protein: 12.5 mg/dl), liver injury (total bilirubin: 1.6 mg/dl), kidney injury (creatinine: 4.8 mg/dl), hyperkalemia (K: 6.3 mEq/L), and hyperglycemia (glucose: 551 mg/dl), but coagulation was normal (platelets: 255 × 10^3^/μl, prothrombin time ratio: 1.1, antithrombin activation: 102%). CSF tests showed that the total protein level was elevated (Table 2). Chest X-ray images showed mild pulmonary edema but not pneumonia. Brain computed tomography (CT) showed mild hydrocephalus and a broken VP shunt tube but not intracranial hemorrhage or mass lesions. Brain magnetic resonance imaging (MRI) showed several old microinfarctions but not acute infarction or inflammation. Her SOFA score was 13, and she was admitted to the ICU with a diagnosis of sepsis, according to the Sepsis-3 criteria. *Enterococcus faecalis* was isolated from her blood and CSF, but not her sputum or urine sampled on the day of admission. The focus of infection could not be identified. Status epilepticus, hyperglycemia, and sepsis-associated encephalopathy were considered possible causes of coma. After starting an antiepileptic drug, seizures did not present during her hospital stay. We started glycemic control with insulin after admission. Her blood glucose was successfully controlled 4 days after initiating insulin. No pathogenic microbes were isolated from her blood and CSF sampled 2 days after admission. Her body temperature returned to normal 4 days after admission. 6 days after admission, she was transferred to the GW because her level of consciousness returned to nearly normal and her vital signs stabilized without oxygen administration or intravenous infusion. Antibiotic treatment was completed in 2 weeks. 40 days after admission, she was transferred to a rehabilitation hospital to increase her ability to perform activities of daily living.

**Table 1:** Orexin levels, treatment, and clinical data.

	**The day of admitting to ICU**	**2 days after admission**	**The day of transferring to GW**	**1 week after antibiotic treatment completion**
Days after admission	0	2	6	20
**Orexin levels**				
Cerebrospinal fluid orexin, pg/ml	Not available	187.3	202.2	285.2
Blood orexin, pg/ml	48.7	57.9	56.8	<40
**Treatments**				
Intravenous infusion, ml/day	2,400	1,440	480	0
Noradrenalin, μg/kg/min	0.15	0.05	0	0
Administration of oxygen, L/min	6	5	1	0
**Clinical assessments**				
SOFA score	13	8	2	1
GCS score	6	12	14	15
CAM-ICU	Not available	+	+	–
**Vital signs**				
Body temperature, °C	37.4	37.3	36.3	36.7
Heart rate, /min	127	83	72	70
Systolic blood pressure, mmHg	94	114	152	134
Diastolic blood pressure, mmHg	53	64	59	76
Mean blood pressure, mmHg	67	80	85	95
Respiratory rate, /min	24	20	17	18

*ICU, intensive care unit; GW, general ward; SOFA, sequential organ failure assessment; GCS, Glasgow coma scale; CAM-ICU, confusion assessment method for the intensive care unit*.

**Table 2:** Laboratory data.

	**The day of admitting to ICU**	**2 days after admission**	**The day of transferring to GW**	**1 week after antibiotic treatment completion**
Days after admission	0	2	6	20
**Atrial blood gas analysis**				
PaCO_2_, mmHg	38.6	39.9	Not tested	Not tested
PaO_2_, mmHg	78.4	98.1	Not tested	Not tested
PaO_2_-FiO_2_ ratio	156.8	245.25	Not available	Not available
Lactate, mmol/L	1.1	1	Not tested	Not tested
**Complete blood counts**				
White blood cells, /μl	24,800	11,600	10,700	5,400
Hemoglobin, g/dl	14.7	11.9	11.1	10.3
Platelet, ×1000/μl	255	160	127	122
**Blood biochemical tests**				
Total bilirubin, mg/dl	1.6	0.5	0.5	Not tested
Creatinine, mg/dl	4.8	1.47	0.94	1.23
Urea nitrogen, mg/dl	117.2	45.9	14.9	10.4
Na, mEq/L	141	154	144	143
K, mEq/L	6.3	4	3.2	3.3
C-reactive protein, mg/dl	12.54	3.91	2.12	0.82
Glucose, mg/dl	551	249	175	115
**Blood coagulation tests**				
Prothrombin time ratio	1.11	1.1	0.95	0.97
Antithrombin activation, %	102	Not tested	Not tested	Not tested
Fibrinogen, mg/dl	774	502	Not tested	Not tested
D-dimer, μg/ml	5.2	4.9	6.6	3.3
**Cerebrospinal fluid tests**				
Total Proteins, /μl	126	28	40	30
Cells, /μl	12	3	0	6

We obtained written informed consent to publish this case report from the patient and next of kin. We measured orexin levels using an orexin radioimmunoassay (Phoenix Pharmaceuticals, Burlingame, CA, USA), which is a standard method for the diagnosis of narcolepsy, at the International Institute for Integrated Sleep Medicine (WPI-IIIS), University of Tsukuba; this center regularly measures orexin levels to diagnose narcolepsy. In healthy humans, CSF orexin levels are detectable by this assay (normal range 250–500 pg/ml), but blood orexin levels are generally below the detection limit (<40 pg/ml) (Makela et al., 2018). In patients with type 1 narcolepsy, CSF orexin levels are <100 pg/ml. In patients with certain pathological conditions of the CNS (e.g., intracranial hemorrhage, head trauma, and Guillain-Barre syndrome), CSF orexin levels are moderately low (range 100–200 pg/ml) (Ripley et al., 2001). As expected, the patient's CSF orexin levels were moderately low (187.3 pg/ml) 2 days after admission but gradually recovered as she recovered from sepsis. CSF orexin level was normal (285.2 pg/ml) 1 week after antibiotic treatment had been completed (Table 1). Unexpectedly, orexin was detected in the blood, although the levels were very low, from the day of ICU admission to the day of transfer to the GW. Blood orexin was not detected 1 week after antibiotic treatment completion (Table 1).

## Discussion

This case showed that the recovery of reduced CSF orexin levels due to systemic inflammation was correlated with recovery from sepsis, supporting the hypothesis that orexin treatment can enhance recovery from sepsis by restoring reduced CSF orexin levels. The detected blood orexin levels were very low and challenging to interpret but may imply that orexin leaks from the CSF into the blood because of BBB dysfunction. Reduced CSF orexin levels could be predicted by the abnormal detection of blood orexin. Further investigations are needed to validate the observations made in this case.

This is the first report investigating orexin levels in the CSF and blood of a patient with sepsis. It is not ethically acceptable in Japan to sample CSF routinely from patients with sepsis by lumbar puncture because it is an invasive medical procedure and can induce meningitis in patients with bacteremia (Teele et al., 1981). Fortunately, we could safely collect CSF samples because the patient had a VP shunt tube. Therefore, the data obtained from this case are rare and provide a new understanding of the pathophysiology of sepsis focused on the dynamics of orexin. On the other hand, as a limitation in this study, the non-functional VP shunt might have affected the patient's orexin levels. The orexin levels returned to normal after recovery from sepsis. Therefore, the impact of the VP shunt on orexin levels might be small, at least under normal conditions. However, we could not completely demonstrate that the VP shunt was not a focus of infection because bacteria were detected in the CSF, although we did not obtain proof of infection in the VP shunt by radio graphical examinations. Additionally, we could not measure the CSF orexin level on the admission day because the CSF sample had already been used for diagnosis and was not stored. However, we showed that the CSF orexin level was clinically low 2 days after admission. Now, we are analyzing the CSF orexin levels on the admission day in patients who were admitted to the Department of Neurology, where lumbar puncture is routinely performed during hospitalization, and finally diagnosed with sepsis.

## Data Availability Statement

The original contributions presented in the study are included in the article/Supplementary Material, further inquiries can be directed to the corresponding author/s.

## Ethics Statement

Ethical review and approval was not required for the study on human participants in accordance with the local legislation and institutional requirements. The patients/participants provided their written informed consent to participate in this study. Written informed consent was obtained from the individual(s) for the publication of any potentially identifiable images or data included in this article.

## Author Contributions

YO: planned, designed, and conducted the work, analyzed the data, and drafted the manuscript. SE: conducted the work. NS: managed the work. SK: supervised the work. All authors contributed to the article and approved the submitted version.

## Funding

This work was supported by the Takeda Science Foundation, the Nakatomi Foundation, and the Japan Society for the Promotion of Science (JSPS) KAKENHI Number JP20K22731.

## Conflict of Interest

The authors declare that the research was conducted in the absence of any commercial or financial relationships that could be construed as a potential conflict of interest.

## Publisher's Note

All claims expressed in this article are solely those of the authors and do not necessarily represent those of their affiliated organizations, or those of the publisher, the editors and the reviewers. Any product that may be evaluated in this article, or claim that may be made by its manufacturer, is not guaranteed or endorsed by the publisher.

## References

[B1] Angus D. C. van der Poll T. (2013). Severe sepsis and septic shock. N. Engl. J. Med. 369, 840–851. 10.1056/NEJMra1208623 23984731

[B2] Cohen I. L. (1993). Definitions for sepsis and organ failure. The ACCP/SCCM consensus conference committee report. Chest 103:656. 10.1378/chest.103.2.656-b 8432190

[B3] Cohen J. (2002). The immunopathogenesis of sepsis. Nature 420, 885–891. 10.1038/nature01326 12490963

[B4] Makela K. A. Karhu T. Jurado Acosta A. Vakkuri O. Leppaluoto J. Herzig K. H. (2018). Plasma orexin-A levels do not undergo circadian rhythm in young healthy male subjects. Front. Endocrinol. 9:710. 10.3389/fendo.2018.00710 30568633 PMC6289979

[B5] Ogawa Y. Irukayama-Tomobe Y. Murakoshi N. Kiyama M. Ishikawa Y. Hosokawa N.. (2016). Peripherally administered orexin improves survival of mice with endotoxin shock. Elife 5:e21055. 10.7554/eLife.21055.018 28035899 PMC5245965

[B6] Ripley B. Overeem S. Fujiki N. Nevsimalova S. Uchino M. Yesavage J.. (2001). CSF hypocretin/orexin levels in narcolepsy and other neurological conditions. Neurology 57, 2253–2258. 10.1212/WNL.57.12.2253 11756606

[B7] Sakurai T. (2007). The neural circuit of orexin (hypocretin): maintaining sleep and wakefulness. Nat. Rev. Neurosci. 8, 171–181. 10.1038/nrn2092 17299454

[B8] Singer M. Deutschman C. S. Seymour C. W. Shankar-Hari M. Annane D. Bauer M.. (2016). The third international consensus definitions for sepsis and septic shock (Sepsis-3). JAMA 315, 801–810. 10.1001/jama.2016.0287 26903338 PMC4968574

[B9] Teele D. W. Dashefsky B. Rakusan T. Klein J. O. (1981). Meningitis after lumbar puncture in children with bacteremia. N. Engl. J. Med. 305, 1079–1081. 10.1056/NEJM198110293051810 6974306

[B10] Widmann C. N. Heneka M. T. (2014). Long-term cerebral consequences of sepsis. Lancet Neurol. 13, 630–636. 10.1016/S1474-4422(14)70017-1 24849863

[B11] Young G. B. Bolton C. F. Austin T. W. (1993). Definitions for sepsis and organ failure. Crit. Care Med. 21:808. 10.1097/00003246-199305000-00031 8482107

